# Comparison of left ventricular systolic function by 2D speckle-tracking echocardiography between normal pregnant women and pregnant women with preeclampsia

**DOI:** 10.15171/jcvtr.2019.50

**Published:** 2019-10-06

**Authors:** Atoosa Mostafavi, Yaser Tase Zar, Farahnaz Nikdoust, Seyed Abdolhossein Tabatabaei

**Affiliations:** Shariati Hospital, Tehran University of Medical Science, Tehran, Iran

**Keywords:** Preeclampsia, 2D Speckle Echocardiography, Cardiac Function

## Abstract

***Introduction:*** In light of previous studies reporting the significant effects of preeclampsia on cardiac dimensions, we sought to evaluate changes in the left ventricular (LV) systolic and diastolic functions in patients with preeclampsia with a view to investigating changes in cardiac strain.

*** Methods:*** This cross-sectional study evaluated healthy pregnant women and pregnant women suffering from preeclampsia who were referred to our hospital for routine healthcare services. LV strain was measured by 2D speckle-tracking echocardiography.

***Results:*** Compared with the healthy group, echocardiography in the group with preeclampsia showed a significant increase in the LV end-diastolic diameter (47.43 ± 4.94 mm vs 44.84 ± 4.30 mm; *P* = 0.008), the LV end-systolic diameter (31.16 ± 33.3 mm vs 29.20 ± 3.75 mm; *P* = 0.008), and the right ventricular diameter (27.93 ± 1.71 mm vs 24.53 ± 23.3; *P* = 0.001). The mean global longitudinal strain was -18.69 ± 2.8 in the group with preeclampsia and -19.39 ± 3.49 in the healthy group, with the difference not constituting statistical significance (*P* = 0.164). The mean global circumferential strain in the groups with and without preeclampsia was -20.4 ± 12.4 and -22.68 ± 5.50, respectively, which was significantly lower in the preeclampsia group (*P* = 0.028).

*** Conclusion:*** The development of preeclampsia was associated with an increase in the right and left ventricular diameters, as well as a decrease in the ventricular systolic function, demonstrated by a decline in global circumferential strain.

## Introduction

Hypertensive disorders of pregnancy are a group of conditions that include pregestational hypertension, gestational hypertension, preeclampsia, and preeclampsia superimposed on chronic hypertension. Preeclampsia is high blood pressure and protein in urine, occurring after week 20 of pregnancy. Preeclampsia is a condition that can threaten maternal and fetal lives during pregnancy. There are studies that show the direct effects of preeclampsia on the left ventricular (LV) mass and other cardiac functional indices.^1^ The cardiovascular system of women during pregnancy is subject to a specific set of physiological changes.^2^ A reduction in systemic vascular resistance leads to a decrease in transfusion and an increase in blood volume, resulting in an increased venous preload. These hemodynamic changes lead to an increase in the thickness of the LV wall, as well as an increase in the size and volume of the LV through cardiac remodeling. However, the systolic and diastolic functions of the LV during a normal pregnancy remain constant with minimal changes.^3,4^ These changes are important for the success of pregnancy, but an additional burden may be imposed on the heart.^5^ In addition, heart disease is the leading cause of maternal mortality during pregnancy, and the number of pregnant women at risk for cardiovascular complications is on the rise. Therefore, the identification and perception of the structure and function of the mother’s heart is of clinical importance and is essential for the management of cardiovascular patients during pregnancy.^6,7^

Despite many reports of maternal heart compatibility, there are discussions about changes in the LV function. Nonetheless, evidence of enlargement in the size of the chambers, as well as the mass and the thickness of the LV wall, is inconsistent.^8^ Previous studies have reported conflicting data on the parameters of the systolic cardiovascular function such as the ejection fraction (EF) and Doppler velocity. These parameters are dependent on the load on the heart; their use is, therefore, limited due to the instability of reported pregnancy outcomes.^9-11^ Recently, 2D echocardiography has shown a significant decrease in longitudinal strain indicators at the end of pregnancy, while the traditional parameters do not reflect these functional changes.^12^ In addition, recent technological advances in 3D spectrum echocardiographic technology have made possible the measurement of all the quantitative and qualitative indicators of the cardiac function that are neglected in the current 2D speckle-tracking echocardiography (2D-STE). 13 Accordingly, given the importance of echocardiography in pregnant women and the timely diagnosis of possible risky conditions, we aimed to determine the normal size, the LVEF, the strain rate, and the LV function during normal pregnancy in healthy women and compare them with pregnant women with preeclampsia.

## Materials and Methods

The present study was a cross-sectional descriptive-analytic study conducted in Shariati Hospital between 2017 and 2018. The study population consisted of healthy and preeclamptic pregnant women who were referred to Doctor Shariati Hospital in order to receive routine healthcare services. In the present study, a healthy pregnancy was considered to be pregnancy in women without any cardiac diseases, preeclampsia, seizure, hypertension, diabetes, or kidney diseases.

The criterion for demonstrating mild preeclampsia was the incidence of hypertension > 140/190 mm Hg after 20 weeks of pregnancy with proteinuria > 300 mg per 24 hours or 30 mg of stable proteinuria (1+ with strip papery) in randomized urine samples. The criteria for the diagnosis of severe preeclampsia were comprised of hypertension > 160/110 mm Hg with proteinuria > 2 g per 24 hours, epigastric pain, headaches, blurred vision, liver enzyme crisis, coagulation disorders, and increased creatinine and blood urea nitrogen.

The exclusion criteria consisted of low imaging quality, high blood pressure, non-pregnancy diabetes, and other abnormal findings such as 1) abnormal dilation in at least one of the 4 cardiac chambers; 2) a left ventricular ejection fraction (LVEF) < 55%, 3) right ventricular (RV) dilatation or hypokinesia, 4) moderate or worse valvular disorders, 4) pericardial effusion, 5) uncorrected cognitive state, 6) congenital heart disease, and 6) diastolic disturbances.

**Figure 1:**
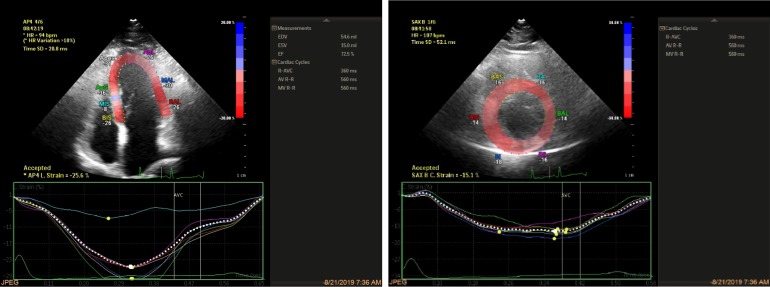
Detection of the endocardial and epicardial border throughout the cardiac cycle (Left: Apical 4 chamber view, right: Parasternal midvantricular short axis view).

**Figure 2:**
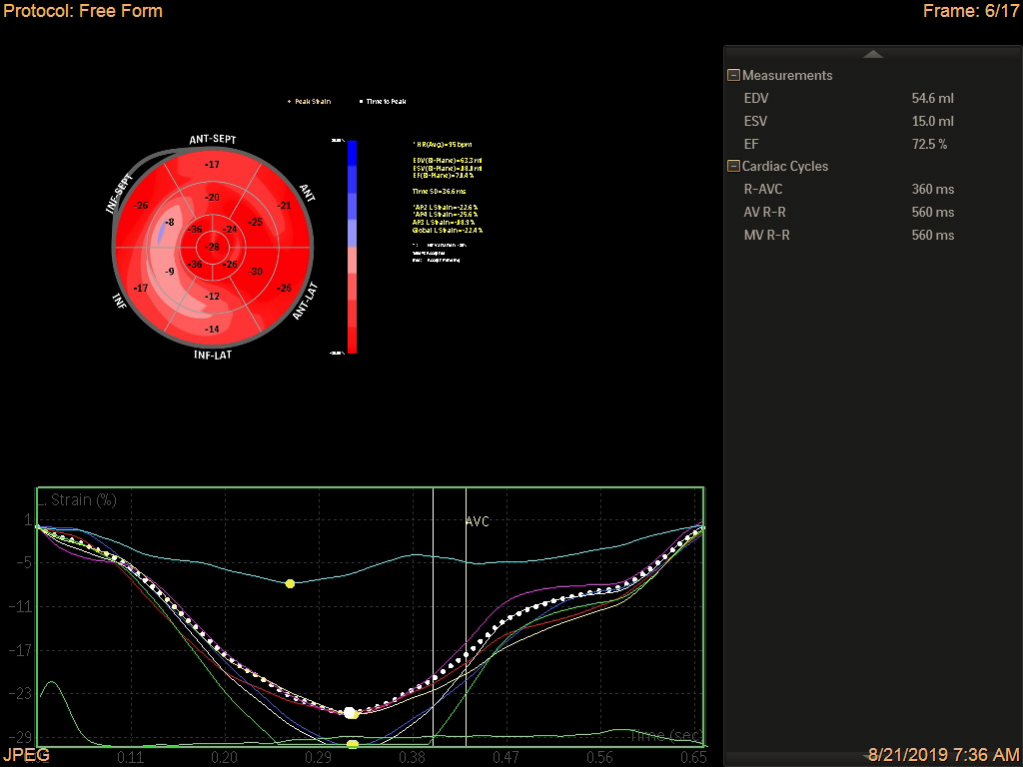
Both strain curve and color coded 17 segments bull’s eye plot.

Sufficient information as regards the study objectives was provided to the patients; and if they consented to participate in the study, the following variables were collected through a self-made questionnaire and echocardiography: age, the LV end-systolic size and volume, the LV end-diastolic volume, the E/E’ ratio, the number of parity, global longitudinal strain, global circumferential strain, the RV peak systolic myocardial velocity by tissue Doppler echocardiography (RVsm), the RV diameter, and tricuspid annular plane systolic excursion (TAPSE). In this study, the third trimester of pregnancy was considered a gestational age of 27 to 42 weeks. Following the assessment and collection of the above data, 2D-STE was conducted with automated cardiac motion quantification(aCMQ) Philips Epic 7 device in the 2 groups of healthy subjects and pregnant women with preeclampsia. The tests used in this study to measure the variables were: 1) simple 2D echocardiography, 2) Doppler echocardiography, and 3) changes in myocardial strain including global longitudinal strain and global circumferential strain. For measurement of 2D strain, first 6 gray scale images were acquired and stored on digital media, then offline software was used to generate the strain data. Images were apical 4 chamber, 2 chamber, 3 chamber for measurement of LV longitudinal strain , and short axis view at basal, mid ventricular and apical view for measurement of circumferential strain. Quality of 2D images were optimal and care was taken to avoid foreshortening. Gray scale frame rate was kept between 30 and 70 frame/sec. ECG was gated and 3 cardiac cycle was acquired for each loop. Automated border detection followed the endocardium and subsequently a correction was manually performed (Figure 1) and strain was automatically calculated by the software expressed as bull’s eye (Figure 2).

All measurement was done by single expert echocardiographist. To reduce interobserver variability, measurements were repeated in random order by an interval of 1 month. To test interobserver variability, a second skilled echocardiographist analyzed data without knowledge of the measurement of first observer.

Descriptive analyses were used to describe the data, including the mean ± the standard deviation (SD) for the quantitative variables and frequencies (percentages) for the categorical variables. The χ2 test, the t-test, and the Mann–Whitney U test were applied to compare the variables. For the statistical analyses, the statistical software IBM SPSS Statistics for Windows, version 22.0, (IBM Corp, released 2013, Armonk, NY) was utilized. A P value < 0.05 was considered statistically significant.

## Results

In the present study, 60 pregnant women with preeclampsia and 40 pregnant women without preeclampsia were enrolled. As is summarized in Table 1 and Table 2, there were no statistically significant differences in regard to the baseline parameters including the mean age, the mean gestational age, the mean body mass index, a history of cigarette smoking, and the number of parity between the 2 groups. The mean LV end-diastolic diameter was significantly higher in the preeclampsia group (47.43 ± 4.94 mm vs 44.84 ± 4.30 mm; *P* = 0.008), the mean LV end-systolic diameter was also significantly higher in the preeclampsia group (31.16 ± 33.3 mm vs 29.20 ± 3.75 mm; *P* = 0.008), and the mean RV diameter was also significantly higher in the preeclampsia group (27.93 ± 1.71 mm vs 24.53 ± 23.3 mm; *P* = 0.001). However, no differences were observed between the 2 study groups vis-à-vis the LV end-diastolic volume, the LV end-systolic volume, the LVEF, RVsm, and TAPSE.

**Table 1:** Basic features of the patients with and without preeclampsia

**Variable**	**Group with preeclampsia**	**Group without Preeclampsia**	***P*** **value**
Mean age, y	33.13 ± 6.57	32.20 ±6.12	0.476
Mean gestational age, wk	33.40 ± 3.54	32.25 ± 4.65	0.164
Mean body mass index, kg/m^2^	31.80 ± 5.44	30.72 ± 4.21	0.290
History of smoking	2 (3.3)	0 (0.0)	0.515
Mean number of parity	2.17 ± 1.28	2.40 ± 1.08	0.345
Number of parity			0.315
One	26 (43.3)	10 (25.0)	
Two	10 (16.7)	12 (30.0)	
Three	16 (26.7)	10 (25.0)	
Four	6 (10.0)	8 (20.0)	
Five	0 (0.0)	0 (0.0)	
Six	2 (3.3)	0 (0.0)	

**Table 2:** Cardiac parameters in the patients with and without preeclampsia

**Variable**	**GroupWithPreeclampsia**	**Group Without Preeclampsia**	***P***
End-diastolic diameter	47.43 ± 4.97	44.80 ± 4.30	0.008
End-systolic diameter	31.16 ± 4.33	29.20 ± 3.75	0.008
End-diastolic volume	89.71 ± 22.45	94.26 ± 17.27	0.280
End-systolic volume	35.19 ± 9.88	38.32 ± 8.38	0.103
LVEF	60.82 ± 5.89	60.14 ± 3.93	0.525
TAPSE	24.97 ± 4.20	25.25 ± 5.06	0.762
RVsm	12.93 ± 1.71	13.10 ± 2.17	0.669
RV diameter	27.93 ± 1.71	24.50 ± 3.23	0.001
Global longitudinal strain	-18.69 ± 2.28	-19.39 ± 3.49	0.164
Global circumferential strain	-20.40 ± 4.12	-22.68 ± 5.50	0.028

LVEF, left ventricular ejection fraction; RV, right ventricle; RVsm, right ventricular peak systolic myocardial velocity.

Regarding the status of strain, the mean global longitudinal strain was -18.69 ± 2.8 in the group with preeclampsia and -19.39 ± 3.49 in the healthy group, with the difference not constituting statistical significance (*P* = 0.164). Nevertheless, the mean global circumferential strain was significantly lower in the preeclampsia group (-20.4 ± 12.4 vs -22.68 ± 5.50; *P* = 0.028). Based on the results, in the patients with preeclampsia, increased systolic diameters, diastolic termination, increased RV diameters, and also decreased global circumferential strain were all expected.

## Discussion

In light of previous studies reporting the significant effects of preeclampsia on cardiac dimensions, as well as the systolic and diastolic functions of the heart, we attempted to evaluate such changes in patients with preeclampsia via an approach to investigate changes in cardiac strain. We found that, firstly, preeclampsia was associated with significant changes in cardiac diameters, with an increase in the LV systolic and diastolic diameters and an increase in the RV diameters. A significant reduction in global circumferential strain was another finding in the current study. It should be noted that although we observed changes in other parameters such as global longitudinal strain and RVsm, these changes were not statistically significant, which may be due to the small sample size of our study or because of term or delayed nature of preeclampsia, and may be this counterbalance changes serve to maintain global ejection fraction.

Changes in the function of the cardiovascular system may be different, depending on the time or severity of preeclampsia, such that in severe or preterm preeclampsia, the changes in cardiac diameters or cardiac output would be much more evident. These categories were not included in our study; nevertheless, the findings of our study chime in with the findings of previous studies.

For instance, Clemmensen et al^12^ also observed no difference in the LVEF between their study groups. Be that as it may, they reported that the LV global longitudinal strain was significantly lower in both early and delayed preeclampsia groups than in the normal group; whereas in our study, the reduction was mainly evident in global circumferential strain and not in the LV global longitudinal strain. Additionally, in their study, Clemmensen and colleagues reported that a severe restriction in ventricular diastolic filling was significantly evident in the early preeclampsia group by comparison with the late preeclampsia group. In our study, this restriction was more evident in the RV. The main advantage of our study over the investigation by Clemmensen et al is that we considered 2 categories of early and late preeclampsia and compared the findings with those in patients in the normotensive status.

Elsewhere, Buddeberg et al^13^ reported diastolic, but not systolic, disturbances after preeclampsia in their study population. Moreover, the authors used 2D-STE and observed a reduction in the LV global strain, endocardial strain, epicardial strain, and longitudinal strain, while we discovered only a reduction in global circumferential strain.

In a study by Vaught et al,^14^ the RV systolic pressure was higher in the preeclamptic group, which is similar to our finding. Nevertheless, what is different is that in their study, the RV longitudinal systolic strain was far less in the preeclampsia group than in the healthy subjects. In an investigation by Yu et al,^15^ patients with preterm hypertension had a much lower LV global longitudinal strain. It is important that during pregnancy and the postpartum period, the patients with preeclampsia in that study had lower radial and global circumferential strain than the healthy controls. In our study, changes in global circumferential strain were significant. The strength of the study by Yu and colleagues is that they followed changes after delivery, which was not possible in our study because many of our patients did not return to our center.

In contrast to our results, Ajmi et al^16^ reported that longitudinal global strain was significantly impaired in their preeclamptic group, although there was no difference between the 2 groups in terms of radial strain and circumferential strain.

With respect to changes in the left- and right-heart dimensions, previous studies have confirmed our findings insofar as in the majority of these investigations, the increase in the LV and RV diameters and in some cases ventricular hypertrophy were quite prevalent in patients with preeclampsia.

In their study, Zangeneh et al^1^ reported that the volume of the LV was significantly higher in their preeclamptic women than in their healthy pregnant women. They also concluded that these changes were due to increased vascular resistance and intravascular volume, leading to an increased cardiac function (increased stroke and increased heart rate) and resulting in an increase in the LV mass. Of course, in severe preeclampsia, hypertrophic changes appear to be due to intravascular resistance and lead to mortality.

Simmons et al^2^ reported that the LV volume and the LVEF in their preeclamptic women were higher than those in their healthy pregnant subjects, which again was more apparent in severe preeclampsia.

Finally, in the context of preeclampsia and the rise in the vascular resistance index, an increase in ventricular dimensions and a significant reduction in ventricular function as measured by global strain can be observed. It is clear that the above changes are more pronounced in severe cases of preeclampsia, wherein we can expect more ventricular hypertrophy and possibly more disruption in ventricular function, followed by yet again a further reduction in the LVEF, which can still be sustained long after delivery.

The limitations of our study are its small sample size and the lack of the follow-up of the patients postpartum.

## Conclusion

In the present study, the occurrence of preeclampsia was associated with an increase in the LV and RV diameters, as well as a decrease in ventricular function in the form of a drop in global circumferential strain. Of course, a decrease in the other indices such as global longitudinal strain and RVsm was also observed; these changes, however, were not significant, which may have been due to our small sample size.

## Competing interests

None.

## Ethical approval

The study was approved by ethical committee of Tehran University of Medical Sciences (No. IR.Tums.MEDICINE.REC.1396.2717).
